# A systematic examination of the use of Online social networking sites for sexual health promotion

**DOI:** 10.1186/1471-2458-11-583

**Published:** 2011-07-21

**Authors:** Judy Gold, Alisa E Pedrana, Rachel Sacks-Davis, Margaret E Hellard, Shanton Chang, Steve Howard, Louise Keogh, Jane S Hocking, Mark A Stoove

**Affiliations:** 1 Centre for Population Health, Burnet Institute, Melbourne, Victoria, Australia; 2 Department of Epidemiology and Preventive Medicine, Monash University, Melbourne, Victoria, Australia; 3 The Nossal Institute for Global Health, The University of Melbourne, Melbourne, Victoria, Australia; 4 Department of Information Systems, The University of Melbourne, Melbourne, Victoria, Australia; 5 Centre for Women's Health, Gender and Society, The University of Melbourne, Melbourne, Victoria, Australia

**Keywords:** Social networking sites, health promotion, sexual health

## Abstract

**Background:**

In recent years social networking sites (SNSs) have grown rapidly in popularity. The popularity of these sites, along with their interactive functions, offer a novel environment in which to deliver health promotion messages. The aim of this paper is to examine the extent to which SNSs are currently being used for sexual health promotion and describe the breadth of these activities.

**Methods:**

We conducted a systematic search of published scientific literature, electronic sources (general and scientific search engines, blogs) and SNSs (Facebook, MySpace) to identify existing sexual health promotion activities using SNSs. Health promotion activities were eligible for inclusion if they related to sexual health or behaviour, utilised one or more SNSs, and involved some element of health promotion. Information regarding the source and type of health promotion activity, target population and site activity were extracted.

**Results:**

178 sexual health promotion activities met the inclusion criteria and were included in the review; only one activity was identified through a traditional systematic search of the published scientific literature. Activities most commonly used one SNS, were conducted by not-for-profit organisations, targeted young people and involved information delivery. Facebook was the most commonly used SNS (used by 71% of all health promotion activities identified), followed by MySpace and Twitter. Seventy nine percent of activities on MySpace were considered inactive as there had been no online posts within the past month, compared to 22% of activities using Facebook and 14% of activities using Twitter. The number of end-users and posts in the last seven days varied greatly between health promotion activities.

**Conclusions:**

SNSs *are *being used for sexual health promotion, although the extent to which they are utilised varies greatly, and the vast majority of activities are unreported in the scientific literature. Future studies should examine the key factors for success among those activities attracting a large and active user base, and how success might be measured, in order to guide the development of future health promotion activities in this emerging setting.

## Background

Social networking sites (SNSs), websites that enable individuals to maintain, form and visualise their social networks [1] - have rapidly become an established part of the online environment. Facebook, Twitter, LinkedIn and MySpace are the most popular SNSs globally [2]. Numerous other SNSs exist, although many are popular only among certain sub-groups or within particular geographic regions [1]. Most SNSs also facilitate public and private messaging, photo, video and other content sharing, provide live updates, enable the formation of groups and organisational pages and include applications such as games, quizzes and polls [1,3-5]. SNSs are part of 'Web 2.0', a loose collection of web-based technologies and services where end-users interact and collaborate as content creators, rather than one-way information flow, which characterises the relatively static websites of 'Web 1.0' [6-8].

Growth in the use of SNSs has been extremely rapid; in August 2010 Facebook reported over 500 million active users, [9] compared to 200 million users in April 2009 [10]. A multi-country study conducted in 2008 found that two thirds of those who use the internet access SNSs [11]. Although young people are the most frequent users of SNSs, use by older adults is increasing [11,12]. The time that individuals spend on SNSs is also increasing; there was a 63% increase in use between 2007 and 2008 compared to an 18% increase in time spent online overall [11]. A 2007 study from the UK reported that 50% of SNS users visit their SNS profile at least every second day [13].

The considerable increase in users of SNSs, their frequency of use, and the interactive functionality of SNSs have prompted calls for health-related interventions, including health promotion, to be delivered in these spaces [8,14-16]. SNSs provide a medium of enormous potential for health promotion both in terms of audience reach and interactive functions that could be exploited for intervention delivery.

In this paper, we examine the current use of SNSs for health promotion. We focus on sexual health promotion, our own area of expertise, and also a critical public health issue where online health promotion interventions are already well established [17-23]. Given the relatively short time in which SNSs have been in use and a lack of consensus with regards to how the outcomes of health promotion activities using SNSs should be evaluated, [8] the aim of this paper is not to assess the impact of individual health promotion activities using SNS, but to provide an overview of existing activities using this medium. This overview identifies the SNSs that are currently being utilised, the organisations responsible for the health promotion activities, and characteristics of the health promotion activities themselves, including an indication of user activity.

## Methods

To examine the use of SNSs for health promotion we developed a novel search strategy covering published scientific literature, electronic sources and SNSs. The search strategy was developed after preliminary searching of published scientific literature revealed very few sexual health promotion activities using SNSs, despite our knowledge of examples from scientific conferences [24-31].

The search strategy developed was informed by previous examples of searching electronic data, [32-35] consultation with a subject librarian and our understanding of SNSs. We experimented with multiple electronic data sources and search terms before developing the final search strategy. All searches were conducted in November 2010.

### Search Strategy

#### 1. Published Scientific Literature

Key medical and scientific databases (CINAHL, Embase, Ovid MEDLINE, PsycINFO, Scopus, Web of Science) were systematically searched. Relevant search terms were developed based on previously published literature [24,36-39]; the full list of search terms used for each database can be found in additional file 1. Search terms for sexual health covered sexual behaviour, sex education, sexually transmitted infections (STIs), condoms and contraception. Search terms for SNSs were adapted from those used by Bardus et al [24] and included social networking (web)sites, online social network(ing) as well as specific SNSs (Facebook and MySpace). These two SNSs were chosen as they are the two most well-established SNSs globally [1]. Where possible, search terms were matched to appropriate subject headings and the 'explode' function used. One screener reviewed the titles and abstracts of all reports retrieved.

#### 2. Electronic Sources

As electronic sources did not permit the same level of complexity in search terms as the medical and scientific databases, simplified search terms were used, adapted from those used for searching the published scientific literature (see additional file 1)

Three types of electronic sources were searched:

**1. General internet search engines: **Google http://www.google.com and Bing http://www.bing.com.

**2. Scientific and medical internet search engines: **Mednar http://www.mednar.com and Scirus http://www.scirus.com.

**3. Blog search engine **- Google blog search http://blogsearch.google.com

As the number of records retrieved by searches of electronic sources is generally unmanageably large, only the first 100 results retrieved from each electronic source for each search term [34] were reviewed for inclusion. Searches were conducted once only, on the same day for each electronic source. One screener reviewed each result retrieved for inclusion.

#### 3. Social Networking Sites

Searches were performed in two key SNSs, Facebook http://www.facebook.com and MySpace http://www.myspace.com. These SNSs do not allow the use of 'AND' or 'OR' operators within searches, so searches used key terms only (see additional file 1).

As with the searches of electronic sources, the first 100 search results for each search term were reviewed for inclusion by one screener.

### Inclusion Criteria

Search results from the published scientific literature, electronic sources and SNSs were included if they met all of the following criteria:

**1. Involved the use of SNS(s)**: SNSs were defined as websites that functioned primarily for individuals to maintain, form and visualise their social networks (consistent with boyd's definition of a SNS) [1]. Websites with other primary functions, such as online dating or content sharing were not included. SNSs could be pre-existing sites, or created specifically for the health promotion activity.

**2. Related to sexual health or behaviour - **Records were included if they involved some information or discussion related to sexual health or behaviour, sexual education, HIV and other sexually transmitted infections, condoms or contraception.

**3. Involved health promotion **- Health promotion was defined as any activity relating to awareness, education, service provision or advocacy related to sexual health or behaviour.

Health promotion activities hosted on multiple websites, including SNSs, were included, as were 'general health' promotion activities on SNSs that included a sexual health focus. Activities that aimed to facilitate communication among professionals were excluded as this communication was not considered a health promotion activity. Records retrieved that were not in English were excluded.

### Data Extraction and Analysis

All records meeting the inclusion criteria were reviewed by viewing the health promotion activity on the SNS used. Information was collected about the organisation responsible for the health promotion activity (name, country of origin, organisation type), the health promotion activity itself (title, year created on SNS, type of SNS) and the content of the health promotion activity (primary sexual health topic, primary target group, purpose of the health promotion activity). The number of end-users (fans/likes/members/followers) of the health promotion activity was also recorded.

As a measure of site activity, we recorded when the most recent post (excluding spam) was made for each health promotion activity. We also recorded the number of posts by the owner and end-users of the health promotion activity in the seven days prior to review of the health promotion activity. 'Likes' on Facebook were considered user posts. No user posts were reported from Twitter because user's posts are not publically displayed on the owners' Twitter profiles. Health promotion activities were defined as 'active' if there were any posts in the month prior to review.

Where the required information could not be sourced from the SNS, reasonable attempts were made to locate the information (for example, visiting the organisation's web page). All details of the health promotion activities were entered into a Microsoft Access 2007 database. Health promotion activities using multiple SNSs were treated as one record.

## Results

### Search Results

**Figure 1:**
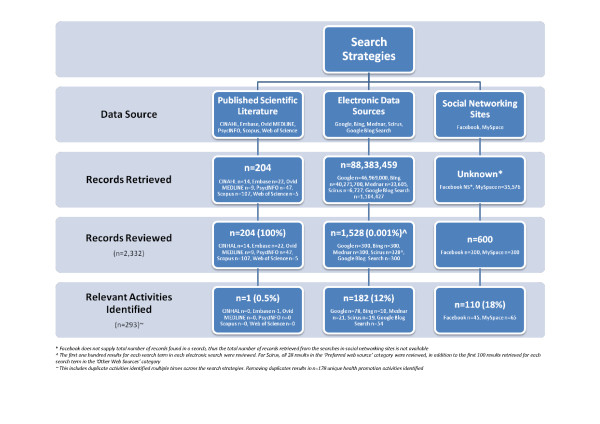
**Search Results**.

Figure 1 displays the number of records retrieved and reviewed using the three search strategies. In total 2332 records were reviewed from the three search strategies; from these records, 293 (13%) health promotion activities were identified that met the inclusion criteria. An additional 27 health promotion activities appeared to meet the inclusion criteria but insufficient information was available to examine them (for example, the presence on a SNS could not be located, or the activity had not yet been conducted). The greatest number of health promotion activities were identified through direct links to SNSs (n = 124, n = 42%) and blogs (n = 55, 19%), followed by news sites (n = 40, 14%). Removal of duplicates resulted in 178 health promotion activities for inclusion (see Additional file 2, table s1).

The search of the published scientific literature identified 18 reports that met the first two inclusion criteria (used SNSs and were related to sexual health and behaviour) but did not meet the third (involved sexual health promotion). Among these 18 excluded reports, 10 examined aspects of SNSs (profiles, groups, networks, posts), [40-49] four reported using SNSs to recruit participants [50-53] and three examined the association between the use of SNSs and sexual health and behaviour [54-56]. One report described an intervention to reduce references to personal sex practices and substance use on publically available user profiles [57].

### Sexual Health Promotion Activities Using Social Networking Sites

#### Social Networking Sites Utilised

Among the 178 health promotion activities identified, 58% used one SNS and 42% used two or more SNSs (Table 1). Facebook was the most commonly used SNS, used by 71% of all health promotion activities. MySpace was used by 46% of activities and Twitter by 30%. Other commercial SNSs used were Ning (n = 3), Bebo (n = 2) and MyMysta (n = 1). Ten health promotion activities used a custom SNS (Table 1).

**Table 1:** Characteristics of Included Health Promotion Activities

Activity Characteristic	Number of Activities (%)
	178 (100)
**Number of SNSs used by activity**	
One	104 (58.4)
Two	50 (28.1)
Three	22 (12.4)
Four	2 (1.1)
**SNS used~**	
Facebook	126 (70.8)
MySpace	82 (46.1)
Twitter	54 (30.3)
Other site^	6 (3.4)
Custom site	10 (5.6))
**Owner**	
Academic institution	20 (11.2)
Collaboration	11 (6.2)
Government	28 (15.7)
Individual	11 (6.2)
Not for profit	77 (43.3)
Private sector	22 (12.4)
Unknown	9 (5.1)
**Country of Origin**	
United States	126 (70.8)
Other	52 (29.2)
**Main Purpose of Activity**	
Connect individuals	10 (5.6)
Campaigns and interventions	51 (28.7)
Organisation/programme presence	112 (62.9)
Unclear/not specified	5 (2.8)
**Sexual Health Focus**	
General health *(including sexual health)*	12 (6.7)
HIV	44 (24.7)
Sexual health	101 (56.7)
STIs *(with/without HIV)*	12 (6.7)
Other*	9 (5.1)
**Target Audience**	
Same-sex attracted individuals	10 (5.6)
People living with HIV	12 (6.7)
Young People	53 (29.8)
Other^+^	16 (9.0)
Unclear/not specified	87 (48.9)

SNS Social Networking Site

~ Not mutually exclusive

^ Includes Ning (n = 3), Bebo (n = 2), MyMsta (n = 1)

* Includes abstinence (n = 3), condoms (n = 3) and those that focused on the health of same-sex attracted individuals (n = 3)

+ Includes females (n = 5), people infected with STIs (n = 4) African Americans (n = 3), males (n = 2), HIV negative individuals (n = 1), Indigenous (n = 1) and sex workers (n = 1)

#### Organisations Responsible

Of the 178 health promotion activities identified, just under half were conducted by not-for-profit organisations (43%), followed by government departments or agencies (16%) the private sector (12%) and academic institutions (11%; Table 1). Fifty-six (32%) of the health promotion activities were conducted by organisations that deliver clinical services (Table 1). Most health promotion activities did not report the year they commenced on SNSs (n = 104, 58%); among those that did, 60 (81%) commenced in 2008 or later.

Health promotion activities were most commonly conducted by organisations or individuals based in the United States (n = 126, 71%), followed by the United Kingdom (n = 20, 11%). Most activities were conducted by organisations or individuals from high income countries; [58] seven were from middle income countries (five from South Africa, one from each of Maldives and Mauritius) and none were low income countries. Two health promotion activities were conducted by multinational organisations while the country of origin of an additional four activities could not be identified.

#### Characteristics of Health Promotion Activities

Among the 178 health promotion activities, three purposes of using SNSs were identified; connecting similar individuals (6%), delivering a campaign or intervention (29%) and having an organisational or programme presence on SNSs (63%; Table 1). Most of the activities focused on sexual health in general (57%) or HIV specifically (25%). Among the 91 activities where the target audience was known, the most common target audience was young people (30% of all activities; Table 1). Three quarters of all health promotion activities (n = 139, 78%) provided information related to sexual health while 87 (49%) provided direct referrals to clinical services.

Table 2 displays the level of site activity for health promotion activities using the three most popular SNSs (Facebook, MySpace and Twitter). The majority of health promotion activities using Facebook (68%) and Twitter (86%) were considered active as there had been new posts within the month prior to review, compared to 21% of health promotion activities using MySpace. The number of end-users and posts in the past seven days varied greatly between health promotion activities. Among the active sites, MySpace had the highest median number of end-users and Twitter the highest median number of owner posts within the seven days prior to review (Table 2). The most active health promotion activities are listed in additional file 3; often, but not always, the most popular activities had the highest numbers of user posts.

**Table 2:** Site Activity among Active^ Health Promotion Activities

	Active		Number of Users		Number of Posts **by Owner**, past seven days*		Number of Posts **by Users**, past seven days*	
	n	%~	Median	Range	Median	Range	Median	Range
Facebook	84	68.3	327	15-111,391	2	0-27	9	0-1,942
MySpace	17	21.3	655	1-20,869	0	0-2	0	0-1
Twitter	44	86.3	565	2-77,087	5	0-195	NA	NA

NA Not available

^ Posts within the 30 days prior to review

~ Denominator is only including health promotion activities where posts could be publically viewed (Facebook n = 126 (98% of all health promotion activities using Facebook), MySpace n = 80 (98%), Twitter n = 51 (94%))

* In the seven days prior to review of the health promotion activity on the SNS used

## Discussion

This study is the first published report describing how SNSs are being used for health promotion, in this case, sexual health promotion. Although there are many examples of SNSs being used for sexual health promotion, most activities are unreported in the scientific literature and the number and activity of end-users varies greatly. Knowing the scale and the scope of the current level of health promotion using SNSs is a key first step in designing more effective health promotion interventions in this new medium.

For the moment, it appears the use of SNSs for sexual health promotion is not widespread: most activities are from the United States, largely target young people and primarily focus on having an organisational or programme presence on SNSs. These outcomes are perhaps not surprising given the emergence of SNSs and the high internet penetration in the United States, the initial young user-base of SNSs [11] and the reality that many organisations may have viewed SNSs (at least initially) as simply an additional online location in which to have a presence, alongside their organisational website. However, as SNSs become more widely used, it is likely that they will also be increasingly used in more diverse ways for health promotion, including for the delivery of campaigns and interventions (now that there is an established user base) and for targeting sub-populations other than young people.

The dominance of three SNSs (Facebook, MySpace and Twitter) within the health promotion activities identified is partly a reflection on our search strategy (which specifically sought out activities on Facebook and MySpace) and also a reflection of the current market share of these SNSs. The advantage of using these established SNSs is that the target audience is already present and interacting with their social networks, unlike creating a custom SNS that must first attract end-users before it can reach individuals for health promotion. However utilising an established SNS can restrict how the health promotion activity is presented, the content that can be provided under each SNS's 'acceptable use' policy, and ownership of online content, which may affect the delivery and fidelity of health promotion activities.

Defining features of Web 2.0 include generation of content by end-users and online social engagement [59]. There was great diversity in popularity and the extent of online interaction among the health promotion activities identified. The most popular health promotion activities had thousands of end-users, with regular posts by owners and end-users each week. Nonetheless, many health promotion activities were inactive, particularly those using MySpace. There seems little purpose in having a relatively 'static' presence on a SNS, with few posts and end-user interactions, in addition to an organisational or campaign 'Web 1.0' website.

From reviewing the health promotion activities identified, it appears that some organisations have simply broadened their online presence into SNSs with relatively minimal effort, using similar content to their existing websites and making little attempt to encourage social activity and engagement. However other organisations appear to have 'purpose built' their presence on SNSs, providing regular updates and delivering content specifically designed for each SNS used. Often, but not always, the most popular sites are also those with the most active online communities. Online social activity does not always happen naturally; [60] future investigations should focus on the most popular and active health promotion activities on SNSs in order to better understand the content, features and approaches that successfully encourage social engagement. These elements could then be used to develop more engaging interventions, which may be more effective as interaction is known to promote deeper learning and understanding [61].

SNSs are constantly evolving. This creates challenges for health promoters, for example when the functionality of SNSs change, or when end-users migrate from one SNS to another. In this review, the high proportion of dormant health promotion activities using MySpace may be a reflection of the more general migration of users from MySpace to Facebook [62-64]. Organisations need to be flexible in responding to this evolution in order to maximise the value of health promotion activities using SNSs. For example, from 2009 Facebook allowed external websites to use Facebook logins and access content from Facebook which has been very popular [65,66]. Thus it is now possible to deliver health promotion activities using functions (and audience reach) of SNSs, without the site actually being hosted on an external commercial platform.

A comprehensive overview of existing sexual health promotion activities using SNSs required us to search electronic sources and SNSs themselves, as well as the published scientific literature. That so little was available in the published scientific literature was most likely a reflection of the rapid emergence and uptake of SNSs, coupled with the time involved in obtaining funding, implementing and evaluating activities using SNSs, and publishing the results. An additional impediment to the scientific publication of health promotion activities using SNSs may be the lack of consensus regarding appropriate evaluation frameworks for these activities [8]. However searches of electronic sources and SNSs bring their own challenges, such as the restricted search capabilities, the inability to replicate searches (see limitations), the incompleteness of information within health promotion activities identified and the unmanageably large number of records retrieved. Given that the need to use electronic sources to produce comprehensive scientific reviews is unlikely to abate, it would be useful to establish 'best practice' guidelines to inform future searches of these contemporary information sources. Such guidelines could include processes for archiving search results for future reference (for example, printing results to PDF).

This review has several limitations. Primarily, the methods for searching electronic sources and SNSs are not well established, and it is likely that some sexual health promotion activities using SNSs were not identified due to the number of search terms and searches possible. As "sexual health" and "health promotion" involve a broad range of topics and activities, we were forced to make choices about which search terms could be used in each data source. However, we attempted to maximise coverage by searching within key SNSs as well as using multiple electronic data sources and multiple search terms. In addition, the searches conducted are not replicable because online content and search algorithms are constantly changing. The search strategy developed also limited the likelihood that campaigns using SNSs primarily for 'viral marketing' would be identified (although one such campaign was identified and included). Only English language sources were searched, which biased results towards health promotion activities from English speaking countries. Due to the large number of records retrieved and time limitations, each record was assessed by only one screener. However the two screeners regularly consulted each other when it was unclear whether the record met the inclusion criteria, and in case of disagreement consulted with a third individual (author AP). For practical reasons, measure of reach was limited to number of end-users of each health promotion activity, while the measure of online social engagement was limited to user posts within a short time period. Although these metrics have clear limitations, it has been argued that online usage statistics are important because they currently offer the one standardised and comparable metric for internet-based interventions and have been associated with positive outcomes across a range of health conditions [8].

This study focused on providing an overview of the current use of SNSs for sexual health promotion; it did not aim to assess the impact of individual health promotion activities. Process and impact evaluations of individual health promotion activities using SNSs should consider inclusion of measures such as:

• Characteristics of end-users - demographics, health knowledge, attitudes and behaviours;

• Quantity of interactions - number of interactions with end-users;

• Quality of interactions - content analysis of interactions to assess relevance and utility;

• Message spread - number of 'shares' and 'retweets' of site content (and characteristics of secondary recipients of site content, if possible);

• Impact of activity on health knowledge, attitudes and behaviour; and

• Cost-effectiveness of activities, particularly in comparison to the cost and effectiveness of delivering health promotion interventions via more traditional channels.

## Conclusion

This investigation presents the first published overview of how SNSs are being used for sexual health promotion. It appears that the call has been heeded; [8,14-16] SNSs *are *being used to deliver health promotion, although these activities have not been described in the published scientific literature or evaluated for their effectiveness in improving health outcomes. The key elements highlighted in this study, such as SNSs used and levels of online social engagement, provide a focal point for individuals and organisations considering using SNSs for health promotion activities. Future studies should consider detailed investigation of individual health promotion activities that have attracted large and active end-user bases in order to elucidate the key factors for success. SNSs offer an unparalleled medium for reaching and engaging with a huge number of individuals; the challenge now is how to maximise the reach and impact of health promotion delivered in this new setting and how to attribute success to the varying intervention components and website functionalities.

## Competing interests

The authors declare that they have no competing interests.

## Authors' contributions

JG contributed to the conception of the manuscript, conducted the literature review and analysis, and was responsible for manuscript preparation and review. AP and RSD contributed to the conception of the manuscript, analysis of the literature and manuscript review. MH contributed to the conception of the manuscript, assisted with literature analysis and manuscript review. SC, SH, LK and JH assisted with literature analysis and reviewed the manuscript. MS contributed to manuscript conception, assisted with literature analysis and reviewed the manuscript. All authors read and approved the final manuscript.

## Pre-publication history

The pre-publication history for this paper can be accessed here:

http://www.biomedcentral.com/1471-2458/11/583/prepub

## Supplementary Material

Additional file 1 **Search terms used**. This file contains the full list of search terms used for each information source, and some additional detail to how the searches of electronic sources were conducted. Click here for file

Additional file 2 **Health promotion activities identified**. This file contains key information about each health promotion activity included in the review. Click here for file

Additional file 3 **Most active health promotion activities**. This files contains a list of the health promotion activities identified with the highest number of users, highest number of posts by owner and highest number of posts by users. Click here for file
