# Supplementary material for: A systematic examination of the use of Online social networking sites for sexual health promotion
Source: BMC Public Health. 2011 Jul 21;11:583. doi: 10.1186/1471-2458-11-583 (PMC3155501; doi:10.1186/1471-2458-11-583)
Supplement: Additional file 1 — Search terms used. This file contains the full list of search terms used for each information source, and some additional detail to how the searches of electronic sources were conducted. [file 1471-2458-11-583-S1.DOC]

# Additional Material: Search Terms Used

## Published Scientific Literature

Note: in all searches, the OR operator was used to combine alternative terms for sexual health and social networking sites (SNS) and the AND operator to combine the sexual health and SNS terms

### CINAHL

#### Sexual Health Terms

1. (TX "Sexually Transmitted Diseases+") OR (TX "Sexually Transmitted Diseases, Bacterial+") OR (TX "Sexually Transmitted Diseases, Fungal+") OR (TX "Sexually Transmitted Diseases, Protozoal+") OR (TX "Sexually Transmitted Diseases, Viral+")
2. (TX "Chlamydia+") OR (TX "Chlamydia Infections+") OR (TX "Chlamydia Trachomatis")
3. (TX "Gonorrhea+")
4. (TX "Syphilis+")
5. (TX "Papillomaviruses") OR (TX "Papillomavirus Infections+")
6. (TX "Herpes Genitalis") OR (TX "Herpes Simplex+")
7. (TX "Human Immunodeficiency Virus+")
8. (TX "Trichomonas Infections+") OR (TX "Trichomonas Vaginitis")
9. (TX "Hepatitis B+")
10. TX "sexual behav*"
11. (TX "Sex+") OR (TX "Safe Sex") OR (TX "Unsafe Sex")
12. (TX "Contraception+") OR (TX "Contraceptives, Postcoital+") OR (TX "Contraceptive Agents, Male") OR (TX "Contraceptives, Oral+") OR (TX "Contraceptives, Oral Combined") OR (TX "Contraceptive Agents+") OR (TX "Contraceptive Devices+") OR (TX "Diaphragms, Contraceptive")
13. (TX "Female Condoms") OR (TX "Condoms")
14. (TX "Coitus")
15. (TX "Sex Education")
16. (TX "Sexual Health")

#### SNS Terms

1. TX "social network* sit*"
2. TX "social network* websit*"
3. TX facebook
4. TX myspace
5. TX "online social network*"

### EMBASE

#### Sexual Health Terms

1. exp sexually transmitted disease/
2. exp CHLAMYDIA/ or exp CHLAMYDIA TRACHOMATIS/
3. exp GONORRHEA/
4. exp SYPHILIS/
5. exp Papilloma virus/
6. exp HERPES SIMPLEX/ or exp GENITAL HERPES/
7. exp Human immunodeficiency virus/
8. exp TRICHOMONAS VAGINALIS/ or exp TRICHOMONAS/
9. exp hepatitis B/
10. exp sexual behavior/
11. exp SEX/ or exp UNSAFE SEX/ or exp SAFE SEX/
12. exp CONTRACEPTION/ or exp ORAL CONTRACEPTION/ or exp BARRIER CONTRACEPTION/ or exp HORMONAL CONTRACEPTION/ or exp EMERGENCY CONTRACEPTION/ or VAGINA CONTRACEPTION/
13. exp CONDOM/
14. exp COITUS/
15. exp sexual education/
16. exp sexual health/

#### SNS Terms

1. social network* sit*.mp. [mp=title, abstract, subject headings, heading word, drug trade name, original title, device manufacturer, drug manufacturer]
2. social network* websit*.mp. [mp=title, abstract, subject headings, heading word, drug trade name, original title, device manufacturer, drug manufacturer]
3. facebook.mp. [mp=title, abstract, subject headings, heading word, drug trade name, original title, device manufacturer, drug manufacturer]
4. myspace.mp. [mp=title, abstract, subject headings, heading word, drug trade name, original title, device manufacturer, drug manufacturer]
5. online social network*.mp. [mp=title, abstract, subject headings, heading word, drug trade name, original title, device manufacturer, drug manufacturer]

### Ovid MEDLINE 1996 to Present with Daily Update

#### Sexual Health Terms

1. exp Sexually Transmitted Diseases/
2. exp Chlamydia Infections/ or exp Chlamydia/ or exp Chlamydia trachomatis/
3. exp Neisseria gonorrhoeae/ or exp Gonorrhea/
4. exp Syphilis/
5. exp Papillomavirus Infections/
6. exp Herpes Genitalis/ or exp Herpes Simplex/
7. exp HIV/ or exp HIV Infections/
8. exp Trichomonas/ or exp Trichomonas Infections/ or exp Trichomonas Vaginitis/ or exp Trichomonas vaginalis/
9. exp Hepatitis B/
10. exp Sexual Behavior/
11. exp Safe Sex/ or exp Unsafe Sex/ or exp Sex/
12. exp Contraception Behavior/ or exp Contraception/ or exp Contraception, Barrier/
13. exp Condoms, Female/ or exp Condoms/
14. exp Coitus/
15. exp Sex Education/
16. sex* health.mp. [mp=title, original title, abstract, name of substance word, subject heading word, unique identifier]

#### SNS Terms

1. social network* sit*.mp. [mp=title, original title, abstract, name of substance word, subject heading word, unique identifier]
2. social network* websit*.mp. [mp=title, original title, abstract, name of substance word, subject heading word, unique identifier]
3. online social network*.mp. [mp=title, original title, abstract, name of substance word, subject heading word, unique identifier]
4. facebook.mp. [mp=title, original title, abstract, name of substance word, subject heading word, unique identifier]
5. myspace.mp. [mp=title, original title, abstract, name of substance word, subject heading word, unique identifier]

### PsycINFO 1806 to October Week 4 2010

#### Sexual Health Terms

1. exp Sexually Transmitted Diseases/
2. chlamydia.mp. [mp=title, abstract, heading word, table of contents, key concepts]
3. exp Neisseria gonorrhoeae/ or exp Gonorrhea/
4. exp Syphilis/
5. exp Human Papillomavirus/
6. exp Herpes Genitalis/
7. exp HIV/ or exp HIV Infections/
8. trichomonas.mp. [mp=title, abstract, heading word, table of contents, key concepts]
9. hepatitis B.mp. [mp=title, abstract, heading word, table of contents, key concepts]
10. exp Sexual Behavior/
11. exp Safe Sex/ or exp Unsafe Sex/ or exp Sex/
12. exp Contraception Behavior/ or exp Contraception/ or exp Contraception, Barrier/
13. exp Condoms, Female/ or exp Condoms/
14. exp Coitus/
15. exp Sex Education/
16. sex* health.mp. [mp=title, abstract, heading word, table of contents, key concepts]

#### SNS Terms

1. social network* sit*.mp. [mp=title, abstract, heading word, table of contents, key concepts]
2. social network* websit*.mp. [mp=title, abstract, heading word, table of contents, key concepts]
3. facebook.mp. [mp=title, abstract, heading word, table of contents, key concepts]
4. myspace.mp. [mp=title, abstract, heading word, table of contents, key concepts]
5. exp Online Social Networks/

### Scopus

(ALL(chlamydia) OR ALL(gonorrhoea) OR ALL(gonorrhea) OR ALL(syphilis) OR ALL(papillomavirus) OR ALL(herpes) OR ALL(hiv) OR ALL(trichomonas) OR ALL("hepatitis B") OR ALL("sexually trans* dis*") OR ALL("sexually trans* infect*") OR ALL("sexual behav*") OR ALL("safe sex") OR ALL("unsafe sex") OR ALL(intercourse) OR ALL(coitus) OR ALL(contraception) OR ALL(condom) OR ALL("sex* education") OR ALL("sex* health")) AND (ALL("social network* sit*") OR ALL("social network* websit*") OR ALL("online social network*") OR ALL(facebook) OR ALL(myspace))

### Web of Science

#### Sexual Health Terms

Topic=(chlamydia) OR Topic=(gonorrhea) OR Topic=(gonorrhoea) OR Topic=(syphilis) OR Topic=(herpes) OR Topic=(hiv) OR Topic=(trichomonas) OR Topic=(hepatitis B) OR Topic=("sexually trans* dis*") OR Topic=("sexually trans* infect*") OR Topic=("sexual behav*") OR Topic=("safe sex") OR Topic=("unsafe sex") OR Topic=(intercourse) OR Topic=(coitus) OR Topic=(contraception) OR Topic=(condom) OR Topic=("sex* education") OR Topic=("sex* health")

#### SNS Terms

Topic=("social network* sit*") OR Topic=("social network* websit*") OR Topic=("online social network*") OR Topic=(facebook) OR Topic=(myspace)

## Electronic Sources

To reduce the likelihood that the previous online activity would alter the results of the searches of electronic sources, the cache was cleared and all web applications closed before conducting each search.

When reviewing the search results, the following rules were followed:

1. If a website would not open a second attempt was made to open the website on another day.
2. If the search results led to the homepage of a website, but the item referred to in the record was not directly accessible from the homepage, an attempt was made to locate the item by searching within the website.
3. If a record retrieved in the search (a primary link) referred to a health promotion activity that potentially met the criteria for inclusion, details of this activity were sourced (a secondary link) and reviewed for inclusion. However, when secondary links referred to additional health promotion activities (tertiary links), these were not followed.

### General Search Engines – Google and Bing

1. “social networking site” AND (“sexually transmitted” OR HIV OR chlamydia OR “sexual behaviour” OR “sexual health”)

2. “facebook” AND (“sexually transmitted” OR HIV OR chlamydia OR “sexual behaviour” OR “sexual health”)

3. “myspace” AND (“sexually transmitted” OR HIV OR chlamydia OR “sexual behaviour” OR “sexual health”)

*Note that Google and Bing were chosen to be searched as they are two of the three most popular search engines globally;[1] Yahoo! Search was not used as it is now powered by Bing.[2]*

### Scientific Search Engines – Mednar

**1. Full Text: "social networking site" AND ("sexually transmitted" OR HIV OR chlamydia OR "sexual behaviour" OR "sexual health")**

2. **Full Text: ("sexually transmitted" OR HIV OR chlamydia OR "sexual behaviour" OR "sexual health") / Title: facebook***

3. **Full Text: ("sexually transmitted" OR HIV OR chlamydia OR "sexual behaviour" OR "sexual health") / Title: myspace***

### Scientific Search Engines – Scirus

1. "social networking site" AND ("sexually transmitted" OR HIV OR chlamydia OR "sexual behaviour" OR "sexual health")

2. title:facebook (("sexually transmitted" OR HIV OR chlamydia OR "sexual behaviour" OR "sexual health"))*

3. title:myspace (("sexually transmitted" OR HIV OR chlamydia OR "sexual behaviour" OR "sexual health"))*

*Note that only results appearing in the ‘Preferred web sources’ and ‘Other web sources’ categories of Scirus were reviewed, as it was expected that records appearing in the ‘Journal sources’ category would have already been retrieved in the search of published scientific literature.*

### Google Blog Search

“social networking site” AND (“sexually transmitted” OR HIV OR chlamydia OR “sexual behaviour” OR “sexual health”)

“facebook” AND (“sexually transmitted” OR HIV OR chlamydia OR “sexual behaviour” OR “sexual health”)

“myspace” AND (“sexually transmitted” OR HIV OR chlamydia OR “sexual behaviour” OR “sexual health”)

## Social Networking Sites

### Facebook, MySpace

1. Sexual health

2. Sexual behaviour^

3. Sexual behavior^

*Note that as Facebook only permits searches by Facebook members, and search results may be influenced by members’ previous activity on Facebook, a user profile with no ‘friends’ or previous ‘likes’ of Facebook pages was used to conduct the search.*

** The searches in Mednar and Scirus restricted mentions of Facebook and MySpace to the title of the record, as using the full text search retrieved many results where the only mention of SNS was a button allowing the user to ‘share’ the contents of the page retrieved via SNS.*

*^ The UK and US spellings of behaviour were not recognised as the same word when searching social networking sites, thus a search for each variant was conducted*

1. **Top Sites: Search Engines**. In.: Alexa. Available at http://www.alexa.com/topsites/category/Top/Computers/Internet/Searching/Search_Engines (accessed 18 Nov 2010); 2010.

2. **Exciting News From Bing and Yahoo!** In.: Bing Search Blog. Available at http://www.bing.com/community/site_blogs/b/search/archive/2010/08/24/exciting-news-from-bing-and-yahoo.aspx (accessed 18 Nov 2010); 2010.
