# Supplementary material for: A systematic examination of the use of Online social networking sites for sexual health promotion
Source: BMC Public Health. 2011 Jul 21;11:583. doi: 10.1186/1471-2458-11-583 (PMC3155501; doi:10.1186/1471-2458-11-583)
Supplement: Additional file 2 — Health promotion activities identified. This file contains key information about each health promotion activity included in the review. [file 1471-2458-11-583-S2.DOC]

Table s1: Health Promotion Activities Identified

| **Title** | **Owner** | **Social Networking Site(s)** | **Country** | **Owner Type** | **Year Created** | **Site Purpose** | **Sexual Health Focus** | **Target Audience** |
| --- | --- | --- | --- | --- | --- | --- | --- | --- |
| #Prevention Revolution | UNAIDS | http://apps.facebook.com/causes/posts/574992?m=15ef63ae; http://twibbon.com/cause/HIV-Prevention-Revolution/Facebook | MULTINATIONAL | Government | 2010 | Campaigns and interventions | HIV | Unclear/ NS |
| A Day with HIV in America | Positively Aware Magazine, Test Positive Aware Network | http://www.facebook.com/group.php?gid=149854168376797&ref=ts; http://twitter.com/A_Day_with_HIV; | USA | Not for profit | 2010 | Campaigns and interventions | HIV | Unclear/ NS |
| ActionAid South Africa | ActionAid International | http://www.facebook.com/group.php?gid=325359665385 | SOUTH AFRICA | Not for profit | 2009 | Organisational presence | HIV | Unclear/ NS |
| Advocates4Youth | Advocates4Youth | http://www.myspace.com/advocatesforyouth; http://www.facebook.com/Advocates4Youth; | USA | Not for profit | 2009 | Organisational presence | SH | Young people |
| AIDS Healthcare Foundation | AIDS Healthcare Foundation | http://www.facebook.com.http.proxy.amoebaos.com/AIDShealth; http://twitter.com/AIDSHealthcare | USA | Not for profit | NS | Organisational presence | HIV | PLWHA |
| Aids in Africa...5700 people die each day.... | Unknown | http://www.myspace.com/6082441 | USA | Individual | NS | Unclear/ NS | HIV | Blacks |
| AIDS.gov | US Government | http://www.facebook.com/AIDS.gov ; http://www.myspace.com/aidsgov; http://twitter.com/AIDSgov; | USA | Government | NS | Organisational presence | HIV | Unclear/ NS |
| AIM HealthCare | Adult Industry Medical (AIM) Health Care Foundation | http://www.myspace.com/aimhealthcare | USA | Not for profit | NS | Organisational presence | SH | Sex workers |
| American Social Health Association | American Social Health Association | http://www.facebook.com/pages/Durham-NC/American-Social-Health-Association/12408621211#!/pages/Durham-NC/American-Social-Health-Association/12408621211?v=wall; | USA | Not for profit | NS | Organisational presence | SH | Unclear/ NS |
| ANDERSON COOPER or Dr. SANJAY GUPTA - PERFORM LIVE HIV TEST ONAIR on 6/27 | Who's Positive | http://www.facebook.com/group.php?gid=130906470267358; | USA | Not for profit | 2010 | Campaigns and interventions | HIV | Unclear/ NS |
| Be One City | Be One City, Inc. | http://www.beonecity.com/ | USA | Unidentified | 2008 | Connect similar individuals | HIV | LGBT/MSM |
| Boston Public Health Commission | Boston Public Health Commission | http://www.facebook.com/HealthyBoston?v=info; http://twitter.com/healthyboston; | USA | Government | NS | Organisational presence | GH | Unclear/ NS |
| Brian tasCHARD | Tasmanian AIDS & Hepatitis Council in Hobart | http://www.myspace.com/185937598 | AUSTRALIA | Individual | NS | Campaigns and interventions | SH | Unclear/ NS |
| Carolina for Kibera - Sexual and Reproductive Health and HIV/AIDS Prevention | Carolina for Kibera | http://www.facebook.com/pages/Carolina-for-Kibera-Sexual-and-Reproductive-Health-and-HIVAIDS-Prevention/130861126944634?v=wall | USA | Not for profit | 2010 | Organisational presence | SH | Unclear/ NS |
| CASH New Orleans | Planned Parenthood Louisiana | http://www.myspace.com/ppconnectingadvocates | USA | Not for profit | NS | Organisational presence | SH | Unclear/ NS |
| CDC | CDC | http://www.myspace.com/cdc_ehealth; http://twitter.com/CDC_eHealth; http://www.facebook.com/CDC | USA | Government | NS | Organisational presence | SH | Unclear/ NS |
| Center for Sexual Pleasure and Health | Centre for Sexual Pleasure and Health | http://www.facebook.com/pages/Pawtucket-RI/The-Center-for-Sexual-Pleasure-and-Health/134984469001 | USA | Not for profit | 2009 | Organisational presence | SH | Unclear/ NS |
| CHAT: Curbing HIV AIDS Transmission | Vista Community Clinic | http://twitter.com/hivchatvcc;http://www.myspace.com/hivchatvcc; http://www.facebook.com/hivchatvcc | USA | Not for profit | NS | Organisational presence | HIV | Young people |
| Check You Out! | NHS Birmingham East and North | http://www.facebook.com/pages/Birmingham-United-Kingdom/CHECK-YOU-OUT/189726708406?ref=ts | UNITED KINGDOM | Government | NS | Campaigns and interventions | STI | Young people |
| Chlamydia Testing (Facebook Advertisements) | NHS Bournemouth and Pool | NA (Facebook advertisements) | UNITED KINGDOM | Government | 2008 | Campaigns and interventions | SH | Young people |
| Choices CAMP (Choices Camp School Project) | California State University | http://www.myspace.com/choicescamp | USA | Academic institution | 2008 | Organisational presence | SH | Young people |
| Citrus County STD/HIV/AIDS Information Specialist | Citrus County Health Department | http://www.myspace.com/citrusstd | USA | Government | 2008 | Organisational presence | SH | Young people |
| Cleveland Department of Public Health | Cleveland Department of Public Health | http://www.facebook.com/pages/Cleveland-OH/Cleveland-Department-of-Public-Health/38975727814?v=info; | USA | Government | NS | Organisational presence | GH | Unclear/ NS |
| Clinicas de Camino Real | Clinicas de Camino Real | http://www.myspace.com/clinicasdelcaminorealinc | USA | Not for profit | NS | Organisational presence | SH | Unclear/ NS |
| CMMB (Catholic Medical Mission Board) | Catholic Medical Mission Board | http://www.facebook.com/CMMBWorldwide; http://twitter.com/CMMBTweets | USA | Not for profit | NS | Organisational presence | GH | Unclear/ NS |
| Condoman | Queensland Association for Healthy Communities | http://www.facebook.com/condoman.is.back | AUSTRALIA | Not for profit | NS | Campaigns and interventions | SH | Indigenous |
| Consider This | Sexual Health and Family Planning ACT | http://www.causes.com/causes/120285-consider-this-ask-your-doctor-for-a-chlamydia-test/about; http://www.shfpact.org.au/considerthis/Consider_Chlamydia/Home.html (project website) | AUSTRALIA | Not for profit | NS | Campaigns and interventions | STI | Young people |
| DailyStrength | DailyStrength | http://www.dailystrength.org/ | USA | Private | 2006 | Connect similar individuals | GH | Unclear/ NS |
| Disparaties in Sexual Health | Unknown (appears to be for a college class) | http://www.facebook.com/pages/Disparities-in-Sexual-Health/131685393543820#!/pages/Disparities-in-Sexual-Health/131685393543820?v=wall | UNKNOWN | Unidentified | 2010 | Campaigns and interventions | SH | Unclear/ NS |
| Dr. Jennifer Berman | Jennifer Berman | http://www.myspace.com/drjenniferberman | USA | Private | NS | Organisational presence | SH | Females |
| Drugs and HIV | National Institute on Drug Abuse | http://www.myspace.com/drugsandhiv; | USA | Government | NS | Campaigns and interventions | HIV | Young people |
| Durnham-Darlington Sexual Health | NHS County Durham & Darlington | http://www.facebook.com/profile.php?id=100000610054314#!/profile.php?id=100000610054314&v=info | UNITED KINGDOM | Government | NS | Organisational presence | SH | Unclear/ NS |
| Elizabeth Boskey's Human Sexual Behavior & STD Education | Elizabeth Boskey | http://www.facebook.com/ElizabethBoskeyPhD; http://twitter.com/About_STD | USA | Individual | NS | Organisational presence | STI | Unclear/ NS |
| EmpowHER | EmpowHER | http://www.facebook.com/empowher; http://twitter.com/empowher; | USA | Private | NS | Organisational presence | GH | Females |
| Fight HIV in DC | DC HIV Working Group, The DC Centre | http://www.facebook.com/group.php?gid=18220526596; http://twitter.com/fighthivindc; http://www.myspace.com/fighthivindc | USA | Not for profit | 2008 | Campaigns and interventions | HIV | Unclear/ NS |
| GCHD: Sexuality Outreach | Genesee County Health Department | http://www.myspace.com/healthy_sexuality | USA | Government | NS | Organisational presence | SH | Unclear/ NS |
| Get Live, Stay Live | ISIS and San Francisco Department of Public Health | http://www.myspace.com/getlivestaylive | USA | Collaboration | NS | Campaigns and interventions | SH | Young people |
| Get Yourself Tested (GYT) | It's Your Sex Life Collaboration | http://www.facebook.com/GYTnow#!/GYTnow?v=info; http://twitter.com/gytnow; | USA | Collaboration | 2009 | Campaigns and interventions | STI | Young people |
| getcluedup | University of Sydney, ReachOut! | http://www.myspace.com/getcluedup; | AUSTRALIA | Collaboration | NS | Campaigns and interventions | STI | Young people |
| GLBTQ Health Headquarters | Unknown | http://www.myspace.com/glbtqhealth | USA | Unidentified | NS | Organisational presence | LGBT health | LGBT/MSM |
| GMFA UK | The Gay Men's Health Charity | http://www.myspace.com/gmfa_uk; http://www.facebook.com/GMFA.UK#!/GMFA.UK?v=wall; http://twitter.com/GMFA_UK | UNITED KINGDOM | Not for profit | NS | Organisational presence | SH | LGBT/MSM |
| Graduate DePaul Advocates for Sexual & Social Health | DePaul University | http://www.facebook.com/pages/Graduate-DePaul-Advocates-for-Sexual-Social-Health/160347333995168 | USA | Academic institution | 2010 | Organisational presence | SH | Young people |
| Greater than AIDS | Kaiser Family Foundation, The Black AIDS Leadership | http://www.facebook.com/greaterthanaids; http://twitter.com/greaterthanaids; | USA | Collaboration | 2009 | Campaigns and interventions | HIV | Blacks |
| Hackney Sexual Health Services | Sexual Health Online for NorthEast London | http://www.myspace.com/hackneysexualhealth;http://www.facebook.com/group.php?gid=55038329047&ref=nf | UNITED KINGDOM | Government | 2009 | Organisational presence | SH | Unclear/ NS |
| Have I got it? | NHS County Durham & Darlington | http://www.facebook.com/pages/Have-I-Got-It/406111305385; | UNITED KINGDOM | Government | NS | Campaigns and interventions | SH | Unclear/ NS |
| Health4Men | Health4Men | http://www.facebook.com/group.php?gid=122081158389 | SOUTH AFRICA | Collaboration | NS | Organisational presence | SH | Males |
| Healthy Gay Cornwell | Cornwall & Isles Of Scilly Primary Care Trust's Health Promotion Service | http://www.facebook.com/group.php?gid=48250246448 | UNITED KINGDOM | Not for profit | NS | Organisational presence | SH | LGBT/MSM |
| HIV Big Deal | Public Health Solutions and New York University | http://www.facebook.com/group.php?gid=23274359391; | USA | Collaboration | 2007 | Campaigns and interventions | STI | LGBT/MSM |
| HIV Campground Project | Unknown | http://www.myspace.com/hiv_campground_project; http://twitter.com/HIV_POZ_CAMP; http://www.facebook.com/pages/HIV-Campground-Project-Worldwide-Support-Group-Friends/218947585626; http://www.hivcampgroundproject.ning.com | USA | Not for profit | 2009 | Organisational presence | HIV | PLWHA |
| HIV Health and Support Network | HIV Health and Support Network | http://www.myspace.com/hivsupportnetwork | USA | Unidentified | NS | Organisational presence | HIV | PLWHA |
| hiv/aidstribe | WebTribes | http://www.hivaidstribe.com/ | USA | Private | 2007 | Connect similar individuals | HIV | PLWHA |
| HIV: What's the Story? | British Red Cross | http://www.facebook.com/group.php?gid=7862676484&v=info; http://www.bebo.com/hivwhatsthestory; http://www.myspace.com/hivwhatsthestory | UNITED KINGDOM | Not for profit | 2007 | Campaigns and interventions | HIV | Young people |
| HIV-911 | Centre for HIV/AIDS Networking, University of KwaZulu-Natal | http://www.facebook.com/home.php#!/group.php?gid=126981516748&v=info; http://twitter.com/hiv911 | SOUTH AFRICA | Academic institution | NS | Organisational presence | HIV | Unclear/ NS |
| HIV-UB2 | Robert Brandon Sandor | http://hiv-ub2.ning.com/; http://twitter.com/SayNO2HIV; http://www.facebook.com/people/Robert-Sandor/565257455#!/profile.php?id=565257455 | USA | Individual | 2010 | Campaigns and interventions | HIV | HIV negatives |
| I practice responsible sexual behavior | Unknown | http://www.facebook.com/group.php?gid=114211628608419#!/group.php?gid=114211628608419&v=info | USA | Individual | 2010 | Campaigns and interventions | SH | Unclear/ NS |
| I Stand With Magic campaign to End Black HIV/AIDS | Magic Johnson Foundation | http://www.facebook.com/group.php?gid=37826337849 | USA | Not for profit | 2008 | Campaigns and interventions | HIV | Young people |
| i-Base | i-Base | http://www.facebook.com/pages/HIV-i-Base/147251881966136?ref=ts; | UNITED KINGDOM | Not for profit | NS | Organisational presence | HIV | PLWHA |
| Illinois Caucus for Adolescent Health | Illinois Caucus for Adolescent Health | http://www.myspace.com/icahil; http://www.facebook.com/illinoiscaucusforadolescenthealth; http://twitter.com/icah | USA | Not for profit | NS | Organisational presence | SH | Young people |
| In The Moment | LA Gay and Lesbian Centre | http://inthemoment.ning.com/; http://twitter.com/inthemomenttv; http://www.facebook.com/pages/IN-THE-MOMENT-HIV-prevention-campaign-at-httpwwwinthemomenttv/119125947522?ref=ts | USA | Not for profit | NS | Campaigns and interventions | SH | LGBT/MSM |
| In*Touch Sexual Health Educators | Mount Holyoke College | http://www.facebook.com/pages/InTouch-Sexual-Health-Educators/310245741414 | USA | Academic institution | 2010 | Organisational presence | SH | Young people |
| Island Sexual Health Society | Island Sexual Health Society | http://www.facebook.com/pages/Island-Sexual-Health/133191143378314?v=info | CANADA | Not for profit | NS | Organisational presence | SH | Unclear/ NS |
| It's Your (Sex) Life | ThinkMTV | http://think.mtv.com/Groups/iysl/ | USA | Private | 2008 | Campaigns and interventions | SH | Young people |
| JCHD Sexual Health | Jackson County Health Department | http://www.myspace.com/jchdsexualhealth | USA | Government | NS | Organisational presence | SH | Young people |
| KIP Education | KIP Education | http://www.facebook.com/pages/KIP-Education-Drugs-Alcohol-Sexual-Health-Education/26091109905; http://twitter.com/KIPEd | UNITED KINGDOM | Private | 2008 | Organisational presence | SH | Young people |
| KISS - Keep It Safe Society | Keep It Safe Student Society | http://www.facebook.com/group.php?gid=35203475996 | MAURITIUS | Academic institution | 2008 | Organisational presence | SH | Young people |
| Knowing Is Sexy | Durham County Health Department | http://www.facebook.com/pages/Durham-NC/Knowing-Is-Sexy/68885537876 | USA | Government | NS | Campaigns and interventions | SH | Unclear/ NS |
| Leeds Sexual Health | NHS Leeds | http://www.facebook.com/profile.php?v=wall&ref=search&id=100000389492763#!/profile.php?id=100000389492763&v=info; http://twitter.com/LeedsSexHealth | UNITED KINGDOM | Government | 2009 | Organisational presence | SH | Unclear/ NS |
| Let's prevent the spread of HIV/AIDS | Unknown | http://groups.myspace.com/index.cfm?fuseaction=groups.groupprofile&groupID=106929854 | USA | Unidentified | 2007 | Campaigns and interventions | HIV | Unclear/ NS |
| LGBT Youth Scotland | LGBT Youth Scotland | http://www.myspace.com/lgbtyouthscotland; http://www.facebook.com/lgbtys | UNITED KINGDOM | Not for profit | NS | Organisational presence | LGBT health | LGBT/MSM |
| Live With It | Incendia Health Studios | http://www.facebook.com/pages/Live-With-It/16451277635; http://www.myspace.com/LWI_invisibleman; http://www.myspace.com/julio_cruceroz; http://www.myspace.com/bobbie_gaines; http://www.myspace.com/trevor_goodman; http://www.myspace.com/isaacmudd | USA | Private | NS | Campaigns and interventions | HIV | Unclear/ NS |
| Living Out Loud: Men of color creating HIV Awareness | The Office of LGBT Student Services, New York University | http://www.facebook.com/event.php?eid=171571102869379 | USA | Academic institution | 2010 | Campaigns and interventions | HIV | Blacks |
| Living Positive by Design | Jack Mackenroth | http://www.myspace.com/livingpositivebydesign | USA | Individual | NS | Campaigns and interventions | HIV | Unclear/ NS |
| LoveLife | LoveLife | http://www.facebook.com/group.php?gid=33983507705; http://twitter.com/loveLifeNGO; http://www.mymsta.mobi/ | SOUTH AFRICA | Collaboration | NS | Organisational presence | HIV | Young people |
| MCDPH Sexual Health | Maricopa County Public Health | http://www.facebook.com/pages/Phoenix-AZ/MCDPH-Sexual-Health/103379423029071#!/pages/Phoenix-AZ/MCDPH-Sexual-Health/103379423029071?v=info | USA | Government | 2010 | Organisational presence | SH | Unclear/ NS |
| MedHelp | MedHelp International | http://www.medhelp.org/ | USA | Private | 1994 | Connect similar individuals | GH | Unclear/ NS |
| Media Advocates for Prevention | Institute of Women & Ethnic Studies | http://www.myspace.com/map_nola; http://www.facebook.com/MAP.IWES; | USA | Not for profit | NS | Organisational presence | SH | Young people |
| Memphis Centre for Reproductive Health | Memphis Centre for Reproductive Health | http://www.myspace.com/reproductivehealth; http://twitter.com/mcrhtn; http://www.facebook.com/memphiscenter | USA | Not for profit | NS | Organisational presence | SH | Unclear/ NS |
| Men & Sexual Health | Unknown | http://www.facebook.com/group.php?gid=135303572667 | UNKNOWN | Individual | 2009 | Campaigns and interventions | SH | Males |
| Metro TeenAIDS | Metro TeenAIDS | http://www.causes.com/causes/54119-metro-teenaids; http://twitter.com/metroteenaids; | USA | Not for profit | NS | Organisational presence | HIV | Young people |
| Morph Monkey Facebook Application | American Social Health Association | http://www.facebook.com/apps/application.php?id=5730937939 | USA | Not for profit | 2008 | Campaigns and interventions | STI | Unclear/ NS |
| Mt. Baker Planned Parenthood | Mt. Baker Planned Parenthood | http://www.myspace.com/444277133;ttp://www.facebook.com/MtBakerPlannedParenthood?ref=ts; http://twitter.com/mbpp; | USA | Not for profit | NS | Organisational presence | SH | Unclear/ NS |
| My Fabulous Disease | Mark S. Kin | http://www.facebook.com/pages/My-Fabulous-Disease/373915927135 | USA | Individual | NS | Campaigns and interventions | GH | Unclear/ NS |
| myGPlife (aka My Generation Pure Life) | WaitWear | http://twitter.com/mygplife; http://www.facebook.com/pages/myGPlife-aka-My-Generation-Pure-Life/72781940180 | USA | Private | 2008 | Organisational presence | Abstinence | Unclear/ NS |
| NAM - the HIV/AIDS Information Charity | NAM Publications | http://www.facebook.com/pages/NAM-the-HIVAIDS-information-charity/99971718192 | UNITED KINGDOM | Not for profit | 2009 | Organisational presence | HIV | PLWHA |
| National AIDS Minority Council | National AIDS Minority Council | http://www.facebook.com/group.php?gid=35281457618; http://twitter.com/NMACCommunity | USA | Not for profit | NS | Organisational presence | SH | Unclear/ NS |
| National Association of People with AIDS (NAPWA) | National Association of People with AIDS (NAPWA) | http://www.facebook.com/napwa; http://twitter.com/napwaus; http://www.myspace.com/napwaus | USA | Not for profit | NS | Organisational presence | SH | Unclear/ NS |
| National Sexuality Resource Center | San Francisco State University | http://www.facebook.com/sexresource; http://www.myspace.com/sexliteracy | USA | Academic institution | NS | Organisational presence | SH | Unclear/ NS |
| National Women's Health Netwok | National Women's Health Netwok | http://www.myspace.com/womensvoice; http://www.facebook.com/people/Helen-Rodriguez-Trias/605237779#!/group.php?gid=2452188397&v=wall; | USA | Not for profit | NS | Organisational presence | SH | Females |
| Netdoctor | Hearst Digital Network, The National Magazine Company Limited (NatMag). | http://www.facebook.com/pages/NetDoctor/136143599746047; http://twitter.com/NetDoctor | UNITED KINGDOM | Private | NS | Organisational presence | GH | Unclear/ NS |
| NYC Teen Mindspace Sexual Health Quiz | NYC Department of Health and Mental Hygiene | http://www.myspace.com/485078065 | USA | Government | NS | Campaigns and interventions | SH | Young people |
| Operation Samahan | Operation Samahan Online | http://www.myspace.com/377440120; http://www.facebook.com/OperationSamahan; http://twitter.com/Y2Y_Center | USA | Not for profit | NS | Organisational presence | GH | Young people |
| Options for Sexual Health BC | Options for Sexual Health | http://www.facebook.com/pages/Options-for-Sexual-Health-BC/163977133618579; http://twitter.com/optbc | CANADA | Not for profit | NS | Organisational presence | SH | Unclear/ NS |
| Options Resource Center for Pregnancy and Sexual Health Education and Counseling | Options Resource Center for Pregnancy and Sexual Health Education and Counseling | http://www.myspace.com/optionspregnancycenter | USA | Not for profit | NS | Organisational presence | SH | Females |
| Options Sexual Health Association | Options Sexual Health Association | http://www.facebook.com/pages/Options-Sexual-Health-Association/64347076574#!/pages/Options-Sexual-Health-Association/64347076574?v=wall | CANADA | Not for profit | NS | Organisational presence | SH | Unclear/ NS |
| Ottawa Youth Sexual Health Coalition | Ottawa County Health Dept, Bethany Chr. Services, teen Moms, Catholic Charities of Westen MI, MI Works, OC Courts, and the FOC. | http://www.myspace.com/ottawacountyhealth; http://www.facebook.com/pages/Holland-MI/Ottawa-Youth-Sexual-Health-Coalition/148538113376; http://twitter.com/oc411 | USA | Collaboration | 2008 | Organisational presence | SH | Young people |
| PatientsLikeMe | PatientsLikeMe | http://www.patientslikeme.com/hiv/community | USA | Private | 2004 | Connect similar individuals | HIV | PLWHA |
| Pima County Health Department STD/HIV Clinic | Pima County Health Department | http://www.myspace.com/pchdsexualhealth; http://twitter.com/pchdsexhealth; | USA | Government | 2009 | Organisational presence | SH | Unclear/ NS |
| Planned Parenthood | Planned Parenthood | http://www.myspace.com/plannedparenthood; http://www.facebook.com/PlannedParenthood; http://twitter.com/ppact; | USA | Not for profit | NS | Organisational presence | SH | Unclear/ NS |
| Planned Parenthood Arizona | Planned Parenthood Arizona | http://www.myspace.com/plannedparenthoodarizona; http://www.facebook.com/plannedparenthoodaz?ref=ts | USA | Not for profit | NS | Organisational presence | SH | Unclear/ NS |
| Planned Parenthood League of Massacheusetts | Planned Parenthood League of Massacheusetts | http://www.myspace.com/plannedparenthoodma; http://www.facebook.com/plannedparenthoodma | USA | Not for profit | NS | Organisational presence | SH | Unclear/ NS |
| Planned Parenthood Mid and South Michigan | Planned Parenthood Mid and South Michigan | http://www.myspace.com/ppecm; http://twitter.com/ppmsm; | USA | Not for profit | NS | Organisational presence | SH | Unclear/ NS |
| Planned Parenthood of Hidalgo County | Planned Parenthood of Hidalgo County | http://www.myspace.com/ppahc; http://www.facebook.com/planithidalgo; http://twitter.com/planithidalgo; | USA | Not for profit | NS | Organisational presence | SH | Unclear/ NS |
| Planned Parenthood of Orange and San Bernardino Counties | Planned Parenthood of Orange and San Bernardino Counties | http://www.myspace.com/plannedparenthoodosbc; http://www.facebook.com/plannedparenthoodosbc | USA | Not for profit | NS | Organisational presence | SH | Unclear/ NS |
| Planned Parenthood of the Heartland | Planned Parenthood of the Heartland | http://www.myspace.com/ppgi; http://www.facebook.com/PPHeartland; | USA | Not for profit | NS | Organisational presence | SH | Unclear/ NS |
| Planned Parenthood of the Southern Finger Lakes | Planned Parenthood of the Southern Finger Lakes | http://www.myspace.com/ppsfl; http://www.facebook.com/group.php?gid=26602603647; | USA | Not for profit | NS | Organisational presence | SH | Unclear/ NS |
| Play It Safe | NHS Bath and North East Somerset | http://www.myspace.com/playitsafecomp | UNITED KINGDOM | Government | 2009 | Campaigns and interventions | SH | Young people |
| Pomegranate Health Collective of Chicago | Pomegranate Health Collective of Chicago | http://www.myspace.com/prhc; http://www.facebook.com/group.php?gid=60771607518; | USA | Not for profit | NS | Organisational presence | SH | Females |
| POS Pride | SafeGuards LGBT Health Resource Center | http://www.myspace.com/pos_pride | USA | Not for profit | NS | Connect similar individuals | HIV | PLWHA |
| Positive Friends | Positive Friends Corp. | http://www.positivefriends.com/ | USA | Private | 2008 | Connect similar individuals | SH | PLWSTIs |
| Positive Singles | SuccessfulMatch | http://www.positivesingles.com/ | USA | Private | 2001 | Connect similar individuals | STI | PLWSTIs |
| Positive Survivors Living with HIV/AIDS | Positive Survivors Living with HIV/AIDS | http://www.facebook.com/pages/Positive-Survivors-Living-With-HIVAIDS/9920013114?v=info; http://twitter.com/PSLWHA | CANADA | Not for profit | NS | Organisational presence | HIV | PLWHA |
| positivefish.com | SucessfulMatch | http://www.positivefish.com/ | USA | Private | NS | Connect similar individuals | STI | PLWSTIs |
| POZ Magazine | Smart + Strong | http://www.facebook.com/POZmagazine; http://twitter.com/pozmagazine; http://www.myspace.com/pozmagazine | USA | Private | NS | Organisational presence | HIV | PLWHA |
| POZIAM | POZIAM | http://www.wix.com/poziam/poziam; http://www.facebook.com/pages/POZIAM-RADIO-SHOW/52894642059?v=info; http://twitter.com/poziam; http://www.myspace.com/poziam | USA | Unidentified | 2007 | Connect similar individuals | HIV | PLWHA |
| PPGNW Teen Clinic | Planned Parenthood Great Northwest | http://www.myspace.com/ppteenclinic; http://www.facebook.com/pages/PPGNW-Teen-Clinic/#!/pages/PPGNW-Teen-Clinic/110984295600082 | USA | Not for profit | NS | Organisational presence | SH | Young people |
| Princeton University Sexual Health Advisors | Princeton University | http://www.facebook.com/pages/Princeton-University-Sexual-Health-Advisors/347389522548 | USA | Academic institution | 2010 | Organisational presence | SH | Young people |
| Project Condom: Season Three | University of South Carolina Share Program | http://www.myspace.com/projectcondom | USA | Academic institution | NS | Campaigns and interventions | Condoms | Young people |
| Project Red Talon | Northwest Portland Area Indian Health Board | http://www.myspace.com/projectredtalon | USA | Not for profit | NS | Campaigns and interventions | SH | Young people |
| RealTalkDC | Metro Teen AIDS | http://www.facebook.com/realtalkdc; http://www.myspace.com/realtalkdc; | USA | Not for profit | NS | Campaigns and interventions | SH | Young people |
| Responsible Sexual Behavior at WVU | Nursing 110 class, West Virginia University | http://www.facebook.com/group.php?gid=142695569343 | USA | Academic institution | 2009 | Campaigns and interventions | SH | Young people |
| Riverside County STD Prevention Program | Riverside County Department of Health | http://www.myspace.com/stdprevention | USA | Government | NS | Organisational presence | STI | Unclear/ NS |
| Rock for AIDS Awareness | Rock Your Awareness | http://www.myspace.com/rockforaids | USA | Not for profit | 2005 | Campaigns and interventions | HIV | Young people |
| Safety Net | Hartford Gay & Lesbian Health Collective | http://www.facebook.com/group.php?gid=83785925055; https://twitter.com/HGLHCSafetyNet | USA | Not for profit | NS | Campaigns and interventions | SH | Unclear/ NS |
| Salford Young Persons Sexual Health | NHS Salford | http://groups.myspace.com/salfordypsexualhealth; http://www.facebook.com/group.php?gid=8209899660; | UNITED KINGDOM | Government | NS | Organisational presence | SH | Young people |
| Sex Talk LIVE Sundays | WBGUFM | http://www.myspace.com/wbgusextalk | USA | Private | NS | Organisational presence | SH | Unclear/ NS |
| Sex, etc | Answer, Rutgers University | http://www.myspace.com/mysexetc; http://twitter.com/sexetc; http://www.facebook.com/SexEtc | USA | Academic institution | NS | Organisational presence | SH | Young people |
| Sexpert | International Planned Parenthood Association | http://apps.facebook.com/areyouasexpert/ | USA | Not for profit | NS | Campaigns and interventions | SH | Unclear/ NS |
| Sexploration with Monika | FCC Free Radio | http://www.myspace.com/sexplorationwithmonika;http://www.facebook.com/pages/Sexploration-with-Monika/98808276430; | USA | Private | NS | Organisational presence | SH | Unclear/ NS |
| SEXTXT | Marie Stopes Australia | http://www.myspace.com/sextxt | AUSTRALIA | Not for profit | NS | Campaigns and interventions | SH | Unclear/ NS |
| Sexual Health & Family Planning Australia | Sexual Health & Family Planning Australia | http://www.facebook.com/shfpa | AUSTRALIA | Not for profit | 2009 | Organisational presence | SH | Unclear/ NS |
| Sexual Health Access Alberta | Sexual Health Acess Alberta | http://www.myspace.com/sexualhealthaccessalberta | CANADA | Not for profit | NS | Organisational presence | SH | Unclear/ NS |
| Sexual Health Advocates | National Coalition for Sexual Health | http://www.facebook.com/SxlHealthAdvocates; http://twitter.com/SexualHealthAdv; http://www.facebook.com/group.php?gid=105819316114646 | USA | Collaboration | 2010 | Organisational presence | SH | Unclear/ NS |
| Sexual Health and Assault Peer Educators (SHAPE) | Northwestern University | http://www.facebook.com/pages/Evanston-IL/SHAPE-Sexual-Health-Assault-Peer-Educators/130645596375 | USA | Academic institution | 2009 | Organisational presence | SH | Young people |
| Sexual Health Awareness and Disease Education (SHADE) | University of Minnesota | http://www.facebook.com/umn.shade?v=info | USA | Academic institution | NS | Organisational presence | SH | Young people |
| Sexual Health Clinic | Unknown | http://www.myspace.com/sexualhealthclinic | UNITED KINGDOM | Unidentified | 2007 | Organisational presence | SH | Unclear/ NS |
| Sexual Health Education | Options Resource Center for Pregnancy and Sexual Health Education and Counseling | http://www.facebook.com/pages/Sexual-Health-Education/368075967414#!/pages/Sexual-Health-Education/368075967414?v=wall | USA | Not for profit | 2010 | Organisational presence | SH | Young people |
| Sexual Health Education Program | University Health Centre, UC Berkeley | http://www.facebook.com/pages/Berkeley-CA/Sexual-Health-Education-Program-SHEP/414856300460#!/pages/Berkeley-CA/Sexual-Health-Education-Program-SHEP/414856300460?v=wall | USA | Academic institution | 2010 | Organisational presence | SH | Unclear/ NS |
| Sexual Health Empowerment Clinic | Midwest Health Centre for Women | http://www.facebook.com/pages/Minneapolis-MN/Sexual-Health-Empowerment-Clinic/142485196767#!/pages/Minneapolis-MN/Sexual-Health-Empowerment-Clinic/142485196767?v=wall | USA | Not for profit | 2009 | Organisational presence | SH | Unclear/ NS |
| Sexual Health Forum | Unknown | http://www.facebook.com/pages/SEXUAL-HEALTH-FORUM/125062628653 | UNKNOWN | Unidentified | 2009 | Campaigns and interventions | SH | Unclear/ NS |
| Sexual Health Forum | National Skin Centre | http://www.facebook.com/pages/Sexual-Health-Forum/121268981261660#!/pages/Sexual-Health-Forum/121268981261660?v=wall; http://www.facebook.com/event.php?eid=105564622840374 | SINGAPORE | Government | 2010 | Organisational presence | STI | Unclear/ NS |
| Sexual Health Information | Unknown | http://www.myspace.com/sexualhealthinformation | USA | Individual | 2009 | Unclear/ NS | SH | Unclear/ NS |
| Sexual Health Peers | University of Illinois | http://www.facebook.com/pages/Sexual-Health-Peers/165994463371#!/pages/Sexual-Health-Peers/165994463371?v=info | USA | Academic institution | 2009 | Organisational presence | SH | Young people |
| Sexual Health Scotland | Healthier Scotland | http://www.facebook.com/pages/Sexual-Health-Scotland/108054947148 | UNITED KINGDOM | Government | 2009 | Organisational presence | SH | Unclear/ NS |
| Sexual Health Singapore | DSC Clinic, Department of STI Control | http://www.facebook.com/pages/Sexual-Health-Singapore/157103270981291 | SINGAPORE | Government | NS | Organisational presence | SH | Unclear/ NS |
| Sexual Health Week | Marie Stopes International | http://www.facebook.com/pages/Sexual-Health-Week/161780754734; http://twitter.com/SexualHealthWk | AUSTRALIA | Not for profit | 2009 | Campaigns and interventions | SH | Unclear/ NS |
| Sexual Health Wymondham | Unknown | http://www.facebook.com/pages/Sexual-Health-Wymondham/110080422353486?v=wall | UNITED KINGDOM | Unidentified | 2010 | Unclear/ NS | SH | Unclear/ NS |
| SHAPE | Women’s Health Teen Clinic, Boulder County AIDS Project | http://www.myspace.com/shaperocks | USA | Collaboration | NS | Organisational presence | SH | Young people |
| Shawn and Gwenn | Shawn and Gwen | http://www.facebook.com/shawndecker; http://twitter.com/shawndecker; http://www.myspace.com/mypetvirus; | USA | Private | NS | Organisational presence | HIV | Young people |
| SHFPACT (Sexual Health & Family Planning ACT) | Sexual Health & Family Planning | http://www.facebook.com/shfpact#!/shfpact?v=info | AUSTRALIA | Not for profit | NS | Organisational presence | SH | Unclear/ NS |
| SHine SA | SHine SA | http://www.facebook.com/pages/Sexual-Health-Information-Networking-and-Education-Inc-SHine-SA/110530312326043 | AUSTRALIA | Not for profit | 2010 | Organisational presence | SH | Unclear/ NS |
| Society for Health Education | Society for Health Education | http://www.facebook.com/group.php?gid=6210555697 | MALDIVES | Not for profit | 2007 | Organisational presence | GH | Unclear/ NS |
| South African Youth Risk Behaviour Survey (YRBS) | South African Medical Research Council | http://www.facebook.com/group.php?gid=107869095921507 | SOUTH AFRICA | Academic institution | 2010 | Organisational presence | SH | Unclear/ NS |
| St John #5/Camp ACE HIV Program | St John Luthern Church | http://www.myspace.com/campacehiv | USA | Not for profit | NS | Organisational presence | HIV | Unclear/ NS |
| Status is Everything | African American Office of Gay Concerns | http://www.facebook.com/StatusIsEverything; http://twitter.com/sienewark | USA | Not for profit | 2010 | Organisational presence | HIV | LGBT/MSM |
| Teen Pregnancy Preventer | Unknown | http://www.myspace.com/teenpregnancypreventer | USA | Individual | 2005 | Unclear/ NS | Abstinence | Young people |
| Teen Sexual Health (TSH) Momentum Team | Multi-agency collaboration | http://www.myspace.com/553619935 | USA | Collaboration | NS | Organisational presence | SH | Young people |
| TeenSource | California Family Health Council | http://www.facebook.com/teensource; http://twitter.com/teensource; | USA | Not for profit | NS | Organisational presence | SH | Young people |
| The Change of Sexuality | Unknown | http://www.myspace.com/471003166 | UNKNOWN | Individual | 2009 | Unclear/ NS | Abstinence | Young people |
| The FaceSpace Project | Burnet Institute | http://www.facebook.com/TheFaceSpaceProject; http://www.facebook.com/JasonHoods; http://www.myspace.com/hoodlum1993; http://www.facebook.com/CharlieAngle; http://www.facebook.com/RicChez; http://twitter.com/RicChez; http://www.facebook.com/EmmaBachet | AUSTRALIA | Academic institution | 2009 | Campaigns and interventions | SH | Young people |
| The Medical Institute for Sexual Health | The Medical Institute for Sexual Health | http://www.myspace.com/medicalinstitute | USA | Academic institution | 2008 | Organisational presence | SH | Young people |
| The Native Youth Sexual Health Network | Native Youth Sexual Health Network | http://www.facebook.com/pages/The-Native-Youth-Sexual-Health-Network/154777717651; http://twitter.com/NYSHN; | USA | Not for profit | NS | Organisational presence | SH | Young people |
| The Power To Be Strong | ActionEqualsLife | http://www.facebook.com/ThePowerToBeStrong; http://twitter.com/PowerToBeStrong; | USA | Not for profit | NS | Campaigns and interventions | HIV | Unclear/ NS |
| The Practice Sexual Health | The Practice | http://www.myspace.com/begoodinbed; http://www.begoodinbed.co.uk/; | UNITED KINGDOM | Private | NS | Organisational presence | STI | Unclear/ NS |
| The Rubber Revolution | District of Columbia Department of Health | http://www.facebook.com/home.php?ref=home#!/pages/Rubber-Revolution-DC/148328615212999; | USA | Government | NS | Campaigns and interventions | Condoms | Unclear/ NS |
| The SafeGuards Project | The Family Planning Council | http://www.myspace.com/safeguards | USA | Not for profit | NS | Organisational presence | GH | LGBT/MSM |
| The Sexual Health Centre | The Sexual Health Centre, Cork | http://www.myspace.com/thesexualhealthcentre; http://www.facebook.com/pages/Cork/The-Sexual-Health-Centre/285489790411?ref=mf; http://twitter.com/SHCCork; http://www.sexualhealthcentre.com/ | IRELAND | Not for profit | NS | Organisational presence | SH | Unclear/ NS |
| The South Carolina Contraceptive Access Campaign | New Mornington Foundation, Advocates for Youth | http://www.myspace.com/502352577; http://www.myspace.com/charlestonyan; http://www.myspace.com/502354066; http://www.myspace.com/502365883 | USA | Not for profit | NS | Campaigns and interventions | SH | Young people |
| The U.S. President's Emergency Plan for AIDS Relief (PEPFAR) | US Government | http://www.facebook.com/PEPFAR; http://twitter.com/uspepfar; | USA | Government | NS | Organisational presence | HIV | Unclear/ NS |
| The Well Project | The Well Project | http://www.facebook.com/thewellproject; http://twitter.com/thewellproject; | USA | Not for profit | NS | Organisational presence | HIV | PLWHA |
| TheBody.com | Health Central | http://www.facebook.com/thebodydotcom; http://twitter.com/TheBodyDotCom | USA | Private | NS | Organisational presence | HIV | Unclear/ NS |
| Trojan Evolve for Sexual Health Awareness | Trojan | http://groups.myspace.com/trojanevolve; | USA | Private | 2007 | Campaigns and interventions | Condoms | Unclear/ NS |
| Turn Facebook RED for 2 weeks for HIV/AIDS Awareness | AIDS Outreach Centre | http://www.causes.com/causes/537134-turn-facebook-red-for-2-weeks-for-hiv-aids-awareness/about | USA | Not for profit | NS | Campaigns and interventions | HIV | Unclear/ NS |
| UNAIDS | UNAIDS | http://www.facebook.com/UNAIDS#!/UNAIDS?v=app_162444257110668; http://twitter.com/UNAIDS | MULTINATIONAL | Government | NS | Organisational presence | HIV | Unclear/ NS |
| Untold Stories | MTV, Viacom | http://www.myspace.com/mtvuntoldstories | USA | Private | 2008 | Campaigns and interventions | HIV | Young people |
| Vista Community Clinic | Vista Community Clinic | http://www.facebook.com/pages/Vista-Community-Clinic/94817677722 | USA | Not for profit | NS | Organisational presence | GH | Unclear/ NS |
| WashU Sexual Health | Accredited Assocciation for Ambulatory Healthcare | http://www.facebook.com/pages/Saint-Louis-MO/WashU-Sexual-Health/321238558896#!/pages/Saint-Louis-MO/WashU-Sexual-Health/321238558896?v=wall | USA | Academic institution | 2010 | Organisational presence | SH | Young people |
| Worlds AIDS Week 2010 UBC | University of British Colombia | http://www.facebook.com/home.php?sk=group_123781784346968&ap=1; | CANADA | Academic institution | 2010 | Campaigns and interventions | HIV | Young people |
| Youth Program @ the Center | San Francisco San Francisco Lesbian Gay Bisexual Transgender (LGBT) Community Center | http://www.myspace.com/sfcenteryouth; http://www.facebook.com/sfcenteryouth; | USA | Not for profit | NS | Organisational presence | LGBT health | LGBT/MSM |

*SH Sexual health generally GH General health STIs Sexually transmitted infections (with/without HIV) NS Not Specified*
