# Supplementary material for: A systematic examination of the use of Online social networking sites for sexual health promotion
Source: BMC Public Health. 2011 Jul 21;11:583. doi: 10.1186/1471-2458-11-583 (PMC3155501; doi:10.1186/1471-2458-11-583)
Supplement: Additional file 3 — Most active health promotion activities. This files contains a list of the health promotion activities identified with the highest number of users, highest number of posts by owner and highest number of posts by users. [file 1471-2458-11-583-S3.DOCX]

# Additional Material: Most Active Health Promotion Activities

| **Highest Number of Users** | **Highest Number of Posts By Owner ^** | **Highest Number of Posts By Users~** |
| --- | --- | --- |
|  | | |
| **Facebook** | | |
| 1. AIDS Healthcare Foundation | 1. Elizabeth Boskey's Human Sexual Behavior & STD Education | 1. Planned Parenthood |
| 1. Planned Parenthood | 1. EmpowHER | 1. AIDS Healthcare Foundation |
| 1. CDC | 1. Planned Parenthood | 1. Greater than AIDS Campaign |
| 1. Greater than AIDS Campaign | 1. Boston Public Health Commission | 1. EmpowHER |
| 1. EmpowHER | 1. POZ Magazine | 1. UNAIDS |
| **MySpace** | | |
| 1. Planned Parenthood | - | - |
| 1. Get Live, Stay Live | - | - |
| 1. Sex, etc | - | - |
| 1. AIDS in Africa....57000 people die each day... | - | - |
| 1. GMFA UK | - | - |
| **Twitter** | | |
| 1. CDC | 1. UNAIDS | - |
| 1. AIDS.gov | 1. Netdoctor | - |
| 1. Sex, etc | 1. EmpowHER | - |
| 1. UNAIDS | 1. Planned Parenthood | - |
| 1. Status is everything | 1. TheBody.com | - |

*^ This was determined by reviewing the number of posts made by owners and users in the seven days prior to review. The health promotion activities using MySpace contained a low number of posts so are not reported. User posts on Twitter are not available (see methods section)*
